# Synergistic Activation of Cardiac Genes by Myocardin and Tbx5

**DOI:** 10.1371/journal.pone.0024242

**Published:** 2011-08-29

**Authors:** Chunbo Wang, Dongsun Cao, Qing Wang, Da-Zhi Wang

**Affiliations:** 1 Department of Cardiology, Children's Hospital Boston, Harvard Medical School, Boston, Massachusetts, United States of America; 2 UNC McAllister Heart Institute, University of North Carolina, Chapel Hill, North Carolina, United States of America; 3 Department of Cell and Developmental Biology, University of North Carolina, Chapel Hill, North Carolina, United States of America; 4 Department of Molecular Cardiology, Cleveland Clinic, Cleveland, Ohio, United States of America; University of Colorado Denver, United States of America

## Abstract

Myocardial differentiation is associated with the activation and expression of an array of cardiac specific genes. However, the transcriptional networks that control cardiac gene expression are not completely understood. Myocardin is a cardiac and smooth muscle-specific expressed transcriptional coactivator of Serum Response Factor (SRF) and is able to potently activate cardiac and smooth muscle gene expression during development. We hypothesize that myocardin discriminates between cardiac and smooth muscle specific genes by associating with distinct co-factors. Here, we show that myocardin directly interacts with Tbx5, a member of the T-box family of transcription factors involved in the Holt-Oram syndrome. Tbx5 synergizes with myocardin to activate expression of the cardiac specific genes atrial natriuretic factor (ANF) and alpha myosin heavy chain (α-MHC), but not that of smooth muscle specific genes SM22 or smooth muscle myosin heavy chain (SM-MHC). We found that this synergistic activation of shared target genes is dependent on the binding sites for Tbx5, T-box factor-Binding Elements (TBEs). Myocardin and Tbx5 physically interact and their interaction domains were mapped to the basic domain and the coil domain of myocardin and Tbx5, respectively. Our analysis demonstrates that the Tbx5G80R mutation, which leads to the Holt-Oram syndrome in humans, failed to synergize with myocardin to activate cardiac gene expression. These data uncover a key role for Tbx5 and myocardin in establishing the transcriptional foundation for cardiac gene activation and suggest that the interaction of myocardin and Tbx5 maybe involved in cardiac development and diseases.

## Introduction

Heart formation is one of the earliest morphogenic events during animal development. The development of the heart is under the control of a complex transcriptional network, and dys-regulation of the expression or genetic mutation of cardiac genes often leads to abnormality in the morphology and the function of the heart [1], [2]. Congenital heart defects (CHD) are found in more than 1% life birth in human beings and are the most common diseases affecting children [3]. One type of CHD, the Holt-Oram syndrome (HOS) occurs in a frequency of 1 of 100,000 with defects in the heart and upper limbs [4]. Mutations of Tbx5, a member of the T-box family of transcription factors, have been identified as a causative gene of the HOS [5], [6]. The Tbx5 mutations found from the HOS patients include missense mutations, deletions that induce open reading frame shifts, and premature truncations [5], [6], [7], [8].

Whereas haploinsufficiency of Tbx5 in transgenic mice mimics the HOS, homologous deletion of the Tbx5 gene led to early embryonic lethality [9]. The fact that deletion of only one copy of the Tbx5 genomic sequence already resulted in defects in the mouse model further suggested that the dosage of the Tbx5 gene is critical for the development of the heart and the expression of Tbx5 downstream genes [10]. Consistent with this view, overexpression of Tbx5 under the β-myosin heavy chain promoter in embryonic mouse hearts led to a failure of ventricular development and embryonic lethality in mice [11]. Overexpression of Tbx5 via gene duplication severely affects the development of the heart and limbs in human patients [12], [13]. Furthermore, carriers of Tbx5 mutations show different levels of impairments on the development of the heart and limbs on an individual basis and it was observed that the severity of defects varies even between patients with the same genetic background [7], [13]. Intriguingly, it was also noticed that some Tbx5 mutations lead to more severe abnormity than others [4]. The Tbx5 point mutation G80R displays more severe defects in the heart, in contrast to that of the R237Q mutation [14]. However, the molecular mechanisms underlying those observations are not fully understood.

Tbx5 cooperates with other transcriptional factors to regulate the expression of its target genes [8]. Tbx5 has been shown to interact with Nkx2.5, GATA4, Mef2c, Sall4 and TAZ to synergistically activate target genes expression in cardiomyocytes [14], [15], [16], [17], [18], [19], [20], [21]. The Tbx5 mutants G80R and R237Q were reported to disrupt its synergy with Nkx2.5 and GATA4 [14]. We have previously found that Tbx5 missense mutations affected its function in activating cardiac gene expression [22].

Myocardin is a potent transcription cofactor for serum response factor (SRF) and is expressed in smooth and cardiac muscles [23]. Myocardin contains no DNA-binding domains and it functions by forming a stable complex with SRF to regulate the expression of cardiac and smooth muscle genes that are under the control of promoters containing the consensus sequence CC(A/T)_6_GG or CArG box [24], [25]. Myocardin is required for smooth muscle gene expression and myocardin knock out mice showed severe defects in smooth cell differentiation, cardiovascular insufficiency and embryonic lethality [26]. Two myocardin homologues, myocardin related transcription factor -A and –B (MRTF-A and -B), are also potent SRF cofactors [27]. Genetic studies revealed that myocardin and MRTFs play critical roles in a variety of biological processes, including vascular smooth muscle development, aortic vessel patterning, mammary myoepithelium formation and others [26], [28], [29], [30], [31], [32]. In addition to SRF, myocardin interacts with several other transcriptional regulators including GATA4, Smad1/3, p300, HDAC4/5, NF-κB, Foxo4 and such interaction modulates the transactivity of myocardin and the expression of the target genes [33], [34], [35], [36], [37], [38]. However, how exactly myocardin discriminates between cardiac and smooth muscle specific genes is still unknown. We hypothesize that myocardin activate cardiac or smooth muscle specific genes by collaborating with distinct transcriptional co-factors. In this study, we report that myocardin physically interacts with Tbx5 to synergistically activate the expression of cardiac genes, but not that of smooth muscle genes.

## Results

### Myocardin and Tbx5 synergistically activate cardiac reporter genes

**Figure 1:**
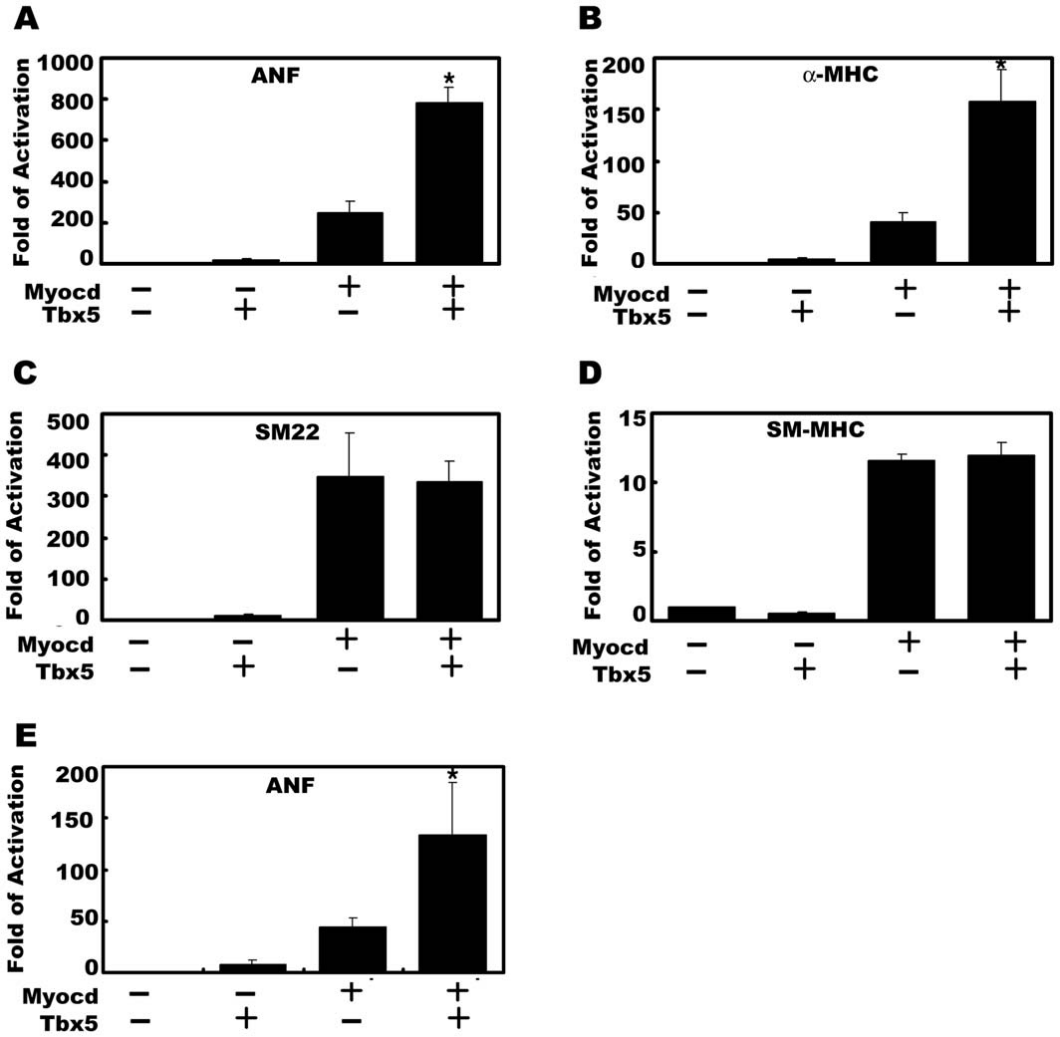
Synergistic activation of cardiac but not smooth muscle genes by myocardin and Tbx5. Luciferase reporters directed by cardiac and smooth muscle gene promoters were co-transfected with myocardin and Tbx5 expression plasmids in COS-7 cells (A-D) or neonatal rat cardiomyocytes (E). (**A**) Synergistic activation of the ANF promoter by myocardin and Tbx5. (**B**) Synergistic activation of the α-MHC promoter by myocardin and Tbx5. (**C**) The effect of myocardin and Tbx5 on the SM22 promoter. Myocardin activates the promoter reporter but there is no synergy between myocardin and Tbx5. (**D**) The effect of myocardin and Tbx5 on the SM-MHC promoter. Myocardin activates the promoter reporter but there is no synergy between myocardin and Tbx5. (**E**) Myocardin and Tbx5 synergistically activate the ANF promoter in neonatal rat cardiomyocytes. In all experiments, the luciferase activity was determined 48 hr after transfection and was presented as fold of activation in which the control was assigned a value of 1. Data represent the mean ± s.d. from at least three independent experiments in duplicate. *P<0.05.

Myocardin potently activates both cardiac and smooth muscle gene expression in a CArG-dependent manner [24]. We hypothesize that myocardin may interact and synergistically cooperate with other cardiac and smooth muscle expressed transcription factors for its distinct function in those two cell types. Previous reports showed that both myocardin and Tbx5 activate the expression of cardiac-specific genes, including ANF [9], [10], [23]. Using luciferase reporters driven by promoters of cardiac or smooth muscle genes, we tested whether myocardin and Tbx5 could synergistically activate these reporter genes in transfected COS-7 cells. As shown in Fig. 1A, both Tbx5 and myocardin activate the ANF luciferase reporter and they synergistically activate the reporter when both proteins were co-transfected together. Note that myocardin is a potent transactivator and activates the reporter gene to a much higher level than that of Tbx5. Similarly, myocardin and Tbx5 synergistically activate a luciferase reporter driven by the promoter of cardiac-specific alpha myosin heavy chain (α-MHC) gene (Fig. 1B). To test whether myocardin and Tbx5 have similar effects on the expression of smooth muscle genes, SM22 and smooth muscle myosin heavy chain (SM-MHC) promoter luciferase reporters were assayed. Consistent with prior reports [23], [39], myocardin but not Tbx5 activates both SM22 and SM-MHC promoter luciferase reporters (Fig. 1C, 1D). However, when both myocardin and Tbx5 were co-transfected with the luciferase reporters, no synergy was observed (Fig. 1C, 1D).

To test whether Tbx5 and myocardin could synergistically activate the ANF promoter in cardiomyocytes, we transfected both myocardin and Tbx5 expression plasmids, together with the ANF luciferase reporter, into primarily cultured neonatal rat cardiomyocytes. As shown in Fig. 1E, whereas both myocardin and Tbx5 can activate the ANF luciferase reporter, their activities were synergized in cardiomyocytes. Together, these results demonstrate that myocardin and Tbx5 synergistically active the expression of cardiac reporter genes but not that of smooth muscle genes.

### The TBE sites of the ANF promoter are required for the synergy between myocardin and Tbx5

**Figure 2:**
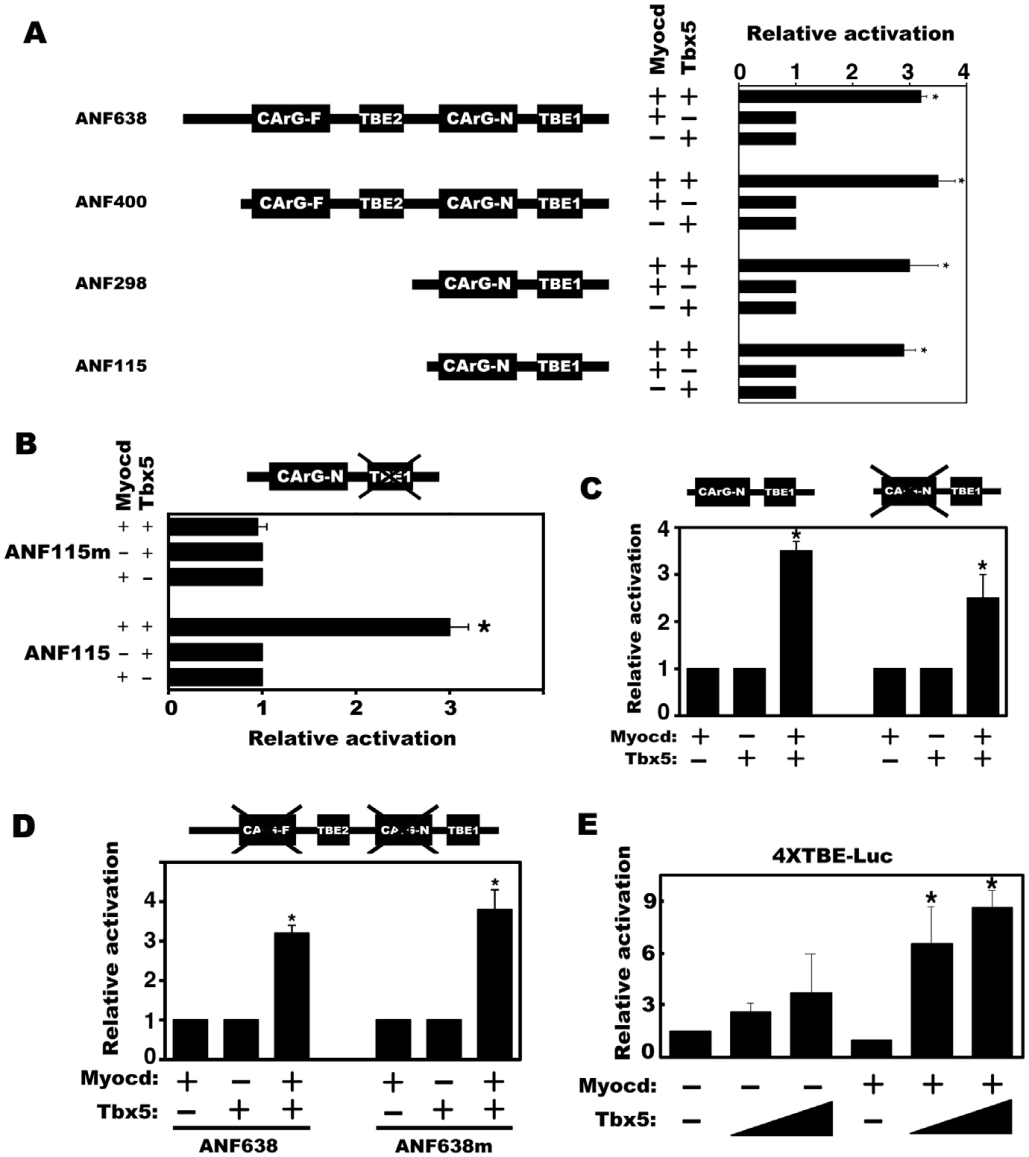
The TBEs are required for the synergistic activation of ANF promoter by myocardin and Tbx5. (**A**) COS-7 cells were transfected with myocardin and Tbx5 expression plasmids and the indicated ANF promoter luciferase reporters in which the CArG boxes and the T-box factor-Binding Elements (TBEs) were indicated, and luciferase activity measured. (**B**) COS-7 cells were transfected with an ANF promoter luciferase reporter (ANF115) or a mutant reporter in which the TBE was mutated (ANF155m) together with myocardin and Tbx5 expression plasmids and luciferase activity measured. (**C**) COS-7 cells were transfected with an ANF promoter luciferase reporter (ANF115) (left three lanes) or a mutant reporter in which the CArG box was mutated (right three lanes) together with myocardin and Tbx5 expression plasmids and luciferase activity measured. (**D**) COS-7 cells were transfected with an ANF promoter luciferase reporter (ANF638) (left three lanes) or a mutant reporter in which both CArG boxes were mutated (ANF638m) (right three lanes) together with myocardin and Tbx5 expression plasmids and luciferase activity measured. (**E**) A luciferase reporter controlled by four tandemly repeats of a consensus Tbx binding elements (TBE) was transfected into COS-7 cells with myocardin and/or Tbx5 expression plasmids and luciferase activity measured. In all the experiments, the luciferase activity was determined 48 hr after transfection and was presented as relative luciferase activity in which the control was assigned a value of 1. Data represent the mean ± s.d. from at least three independent experiments in duplicate. *P<0.05.

The promoter/enhancer of the ANF gene contains multiple cis-elements for several transcription factors, including that of Nkx2.5-binding site (NKE), MEF2-binding site (MEF), GATA-element (GATA), T-box factor-Binding Element (TBE) as well as SRF-binding site (CArG) [1], [9]. To define the cis-elements of the ANF promoter that are responsible for the synergy of myocardin and Tbx5, luciferase reporters driven by truncated ANF promoter sequences were tested in luciferase reporter assays (Fig. 2A). There are two CArG boxes, CArG-F and CArG-N, and two TBEs, TBE2 and TBE1, on the promoter/enhancer of the ANF gene. Analysis of a series of ANF promoter deletion mutations revealed a decrease in the overall potential of myocardin or Tbx5 to activate the reporter gene. However, myocardin and Tbx5 still displayed synergy on the minimal ANF promoter (ANF115) in which a single CArG and TBE remain (Fig. 2A), suggesting that these cis-elements are sufficient to mediate the synergy of myocardin and Tbx5 on the ANF promoter reporter gene.

Next, we mutated the TBE site from the ANF115 minimal promoter and tested the activities of myocardin and Tbx5 to activate the reporter gene. Interestingly, we found that the TBE mutation abolished the synergy between myocardin and Tbx5 to activate this reporter (Fig. 2B). Conversely, we mutated the CArG box from the ANF 115 minimal promoter and found that the CArG mutation did not completely abolish the synergy between myocardin and Tbx5, though their overall activity was significantly decreased (Fig. 2C). To further define the cis-element requirement for myocardin and Tbx5 synergy, we mutated both CArG boxes from the ANF638 promoter and test their activation by myocardin and Tbx5. Interestingly, myocardin and Tbx5 were able to synergistically activate this mutant promoter (Fig. 2D), indicating that the TBE sites were sufficient to mediate the synergy of myocardin and Tbx5. This notion was further supported by the observation that myocardin and Tbx5 were able to synergistically activate a luciferase reporter that is driven by tandem repeats of four TBEs (Fig. 2E). Together, our data suggest that the T-box factor-Binding Elements (TBEs) are essential for the synergy of myocardin and Tbx5 to activate the promoter of the cardiac gene ANF.

### Myocardin and Tbx5 directly interact

**Figure 3:**
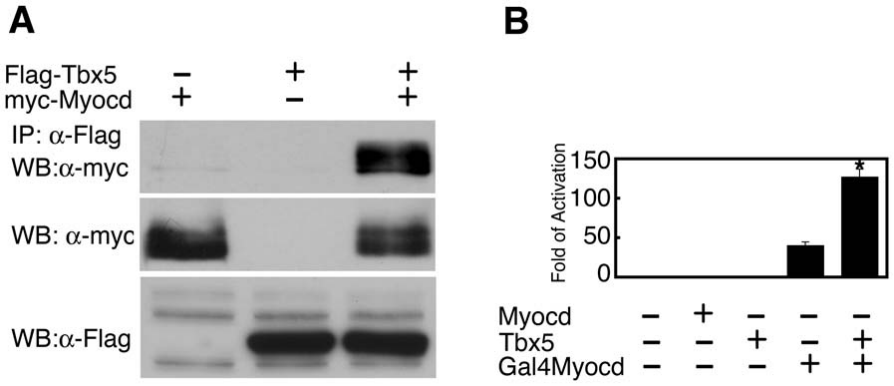
Myocardin and Tbx5 interact directly. (**A**) COS-7 cells were transfected with expression plasmids encoding Flag-tagged Tbx5 and Myc-tagged myocardin as indicated. Tbx5 was immunoprecipitated (IP) by anti-Flag antibodies, and anti-Myc antibodies were used to detect the presence of myocardin in the immunoprecipitates by Western blot (WB) analysis (upper panel). One-fifteenth of cell extracts were directly Western blotted (WB) to detect the presence of myocardin and Tbx5 proteins by anti-Myc or anti-Flag antibodies, respectively (middle and lower panels, respectively). (**B**) An expression plasmid encoding full-length myocardin (1-935) fused to GAL4 DNA binding domain (Gal4Myocd) and the pL8G5-luciferase reporter were transfected in the absence or presence of Tbx5 expression plasmid into COS-7 cells and luciferase activity was determined. pcDNA-Myocardin (Myocd) and pcDNA-Tbx5 (Tbx5) were used as controls. Luciferase activity was determined 48 hr after transfection and was presented as fold of activation in which the control was assigned a value of 1. Data represent the mean ± s.d. from at least three independent experiments in duplicate. *P<0.05.

To test whether Tbx5 and myocardin interact directly, co-immunoprecipitation (Co-IP) assays were performed with cell extracts in which Myc- or Flag-tagged myocardin and Tbx5 were overexpressed in COS-7 cells. When Flag-Tbx5 was precipitated using anti-Flag antibodies, Tbx5-associated Myc-myocardin was readily detected by Western blotting (Fig. 3A). No such interaction was detected when either Flag-Tbx5 or Myc-myocardin was overexpressed alone, demonstrating the specificity of the Co-IP assays.

We used an alternative approach to confirm the interaction between myocardin and Tbx5. The full-length sequence of the myocardin gene was fused with the DNA-binding domain of the Gal4 gene to create a Gal4-myocardin fusion protein (Gal4Myocd). The Gal4-myocardin was then transfected with the pL8G5-luciferase reporter in which the luciferase reporter is under the control of the Gal4 binding element UAS. Gal4-myocardin, but not pcDNA-Tbx5 or pcDNA-myocardin, potently activated the reporter gene (Fig. 3B). Addition of a Tbx5 expression plasmid further enhanced myocardin activity, indicating that Tbx5 likely interacted with myocardin directly in this setting (Fig. 3B).

### Map the interaction domains of myocardin and Tbx5

**Figure 4:**
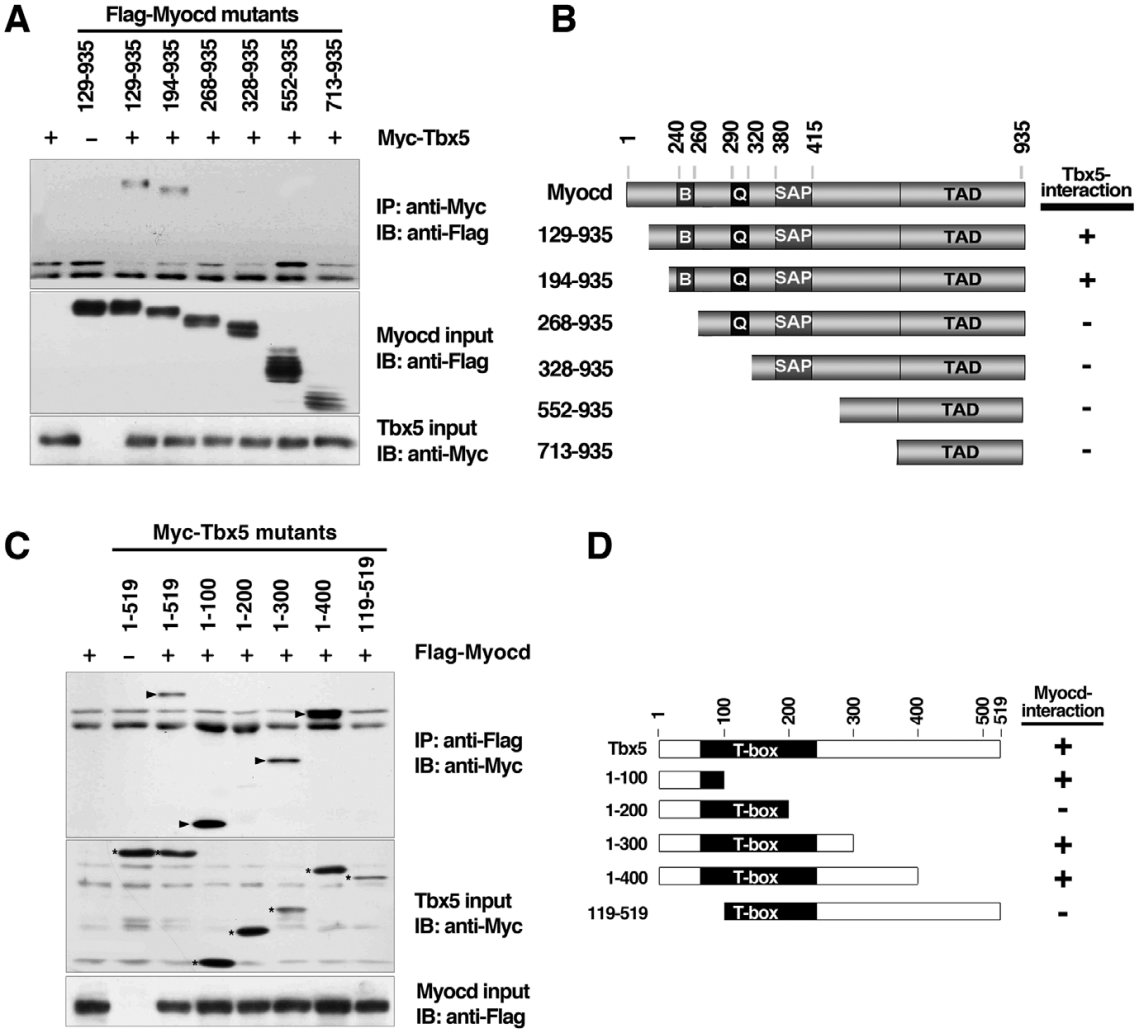
Mapping of the myocardin and Tbx5 interaction domains. (**A**) COS-7 cells were transfected with expression plasmids encoding Myc-tagged Tbx5 and a collection of Flag-tagged myocardin deletion mutants. Tbx5 was immunoprecipitated (IP) by anti-Myc antibodies, and anti-Flay antibodies were used to detect the presence of myocardin in the immunoprecipitates by immunoblot analysis (IB) (upper panel). One-fifteenth of cell extracts were directly immunoblotted (IB) to detect the presence of myocardin and Tbx5 proteins by anti-Flag or anti-Myc antibodies, respectively (middle and lower panels, respectively). (**B**) A schematic summary of myocardin and Tbx5 protein interaction domains. Myocardin domains abbreviated as follows: B, basic domain; Q, a stretch of glutamine residues; SAP, SAF A/B, Acinus, PIAS domain; TAD, transactivation domain. (**C**) COS-7 cells were transfected with expression plasmids encoding Flag-tagged myocardin and a collection of Myc-tagged Tbx5 deletion mutants. Myocardin was immunoprecipitated (IP) by anti-Flag antibodies, and anti-Myc antibodies were used to detect the presence of Tbx5 in the immunoprecipitates by immunoblot (IB) analysis (upper panel). One-fifteenth of cell extracts were directly immunoblotted (IB) to detect the presence of Tbx5 and myocardin proteins by anti-Myc or anti-Flag antibodies, respectively (middle and lower panels, respectively). (**D**) A schematic summary of myocardin and Tbx5 protein interaction domains.

To map the domains of myocardin that are responsible for its interaction with Tbx5, truncated myocardin mutants were generated and their potential interaction with Tbx5 was examined by co-immunoprecipitation (Co-IP) assays. The truncation mutants from the N-terminus up to the basic domain of myocardin can still interact with Tbx5, while further deletion past the basic domain abolished this capability (Fig. 4A). These results indicate that the regions between amino acid (aa) 195-267 are required for myocardin to interact with Tbx5 (Fig. 4A, 4B). To further test whether the basic domain is critical for the interaction, Co-IP experiments were performed using a myocardin deletion mutant lacking only the basic domain [23]. Indeed, the basic domain deletion myocardin mutant failed to interact with Tbx5 (data not shown). These results suggest that the basic domain of myocardin is required for myocardin to mediate its interaction with Tbx5.

Next, we attempted to map the domains of the Tbx5 protein that mediate its interaction with myocardin. Series of Tbx5 deletion mutants were generated to test their interaction with myocardin using co-immunoprecipitation (Co-IP) assays. Our results showed that the N-terminal region (aa 1-100) was sufficient to interact with myocardin (Fig. 4C, 4D). Similarly, the constructs (aa 1-300) and (aa 1-400) also interacted with myocardin. Surprisingly, we found that regions between aa 1-200 failed to interact with myocardin (Fig. 4C, 4D). This observation is surprising because a shorter region (aa 1-100) of the Tbx5 protein was already sufficient to mediate its interaction with myocardin. One possible reason is that a different conformation is adopted in the aa 1-200 fragment; alternatively the region between aa 101-200 may inhibit the interaction between these two proteins. Consistent with the notion that the N-terminus (aa 1-100) was sufficient to mediate myocardin interaction, a N-terminal deletion mutant (aa 119-519) lacking this region abolished its myocardin interaction (Fig. 4C, 4D). Together, these results define the N-terminal region of the Tbx5 protein as its myocardin interaction domain.

### Myocardin and Tbx5 do not form a stable ternary complex on DNA elements

**Figure 5:**
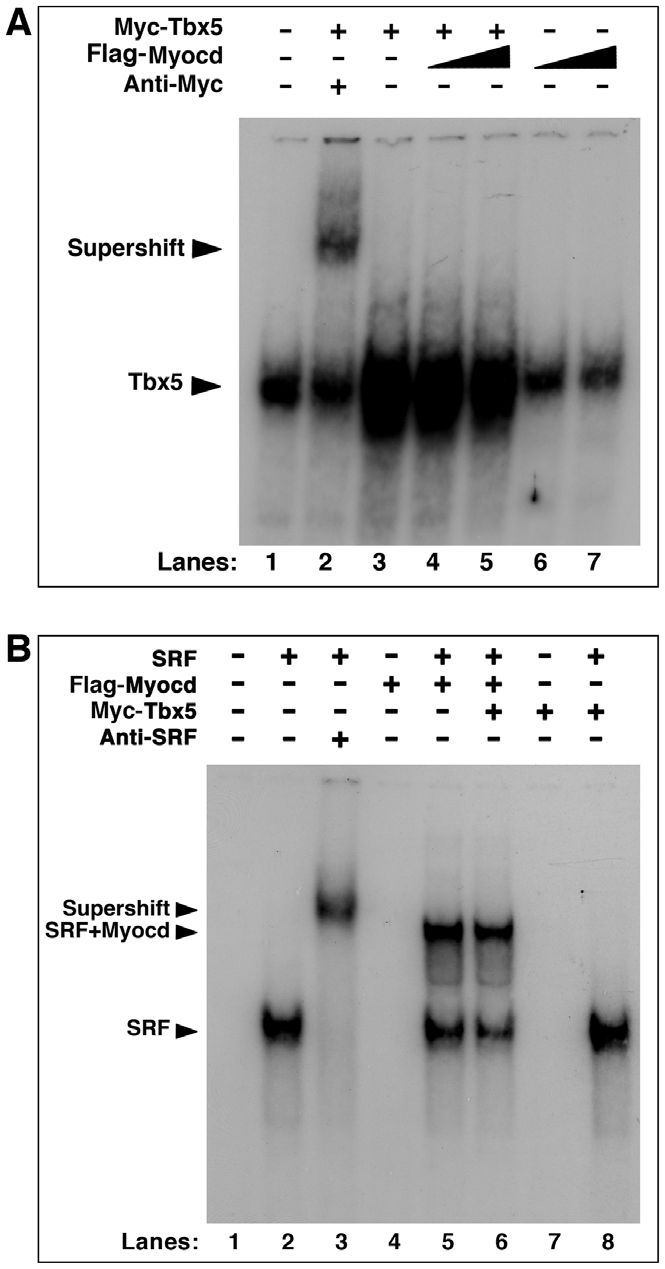
Tbx5 and myocardin do not form a stable ternary complex on CArG-box or TBE. Myc-tagged Tbx5 and Flag-tagged myocardin proteins were expressed by *in vitro* transcription and translation and incubated with radiolabeled probes. (**A**) Electrophoresis mobility shift assays were performed with a ^32^P-labeled TBE probe for Myc-tagged Tbx5 in the presence and absence of increasing amount of Flag-myocardin. Anti-Myc antibodies were applied for supershift. The Tbx5/TBE complexes and the supershift complex were indicated. (**B**) Electrophoresis mobility shift assays were performed with a ^32^P-labeled CArG probe for Flag-myocardin in the presence and absence of SRF, or in the presence and absence of Myc-Tbx5. Anti-SRF antibodies were applied for supershift. The SRF/CArG complexes (SRF), the Myocd/SRF/CArG ternary complexes (SRF+Myocd) and supershift complex (Supershift) were indicated.

Myocardin associates with the CArG boxes via the formation of a CArG/SRF/myocardin ternary complex [23]. In contrast, Tbx5 directly binds to the TBE sequence on the promoter of its target genes as a monomer [9], [14]. We first tested whether myocardin affects the binding of Tbx5 to TBE, or myocardin could even form a ternary complex with Tbx5/TBE. Electrophoretic motility shift assays (EMSAs) were performed using the conserved TBE sequences as probes. When Myc-Tbx5 proteins were included with the probe, there was a very strong band formation (lane 3 in Fig. 5A), indicating the formation of Tbx5/TBE complex. Noticeably, there is a non-specific band co-migrating to the same position as the Tbx5/TBE band, evidenced by its presence in the control lane in which no proteins were added (lane 1 in Fig. 5A). The specificity of the Tbx5/TBE complex is further supported by the formation of “supershift” when Myc antibodies were added into the reaction (lane 2 in Fig. 5A). Addition of myocardin proteins did not substantially affect the formation of the Tbx5/TBE complex, neither did any new band appear (lanes 4–5, Fig. 5A), suggesting that myocardin does not have a direct effect on the formation of Tbx5/TBE complex. Myocardin proteins by themselves did not bind to the TBE probe in the assay (lanes 6-7, Fig. 5A).

Next, we performed EMSAs to test whether Tbx5 could affect the formation of myocardin/SRF/CArG ternary complex. When SRF was incubated with the CArG box probe, they form CArG/SRF complex (lane 2, Fig. 5B). Inclusion of the anti-SRF antibodies in the reaction led to a slower migrating band, representing the CArG/SRF/antibody “supershift”, demonstrating the specificity of protein-DNA complex (lane 3, Fig. 5B). Inclusion of myocardin and SRF resulted the formation of myocardin/CArG/SRF ternary complex (lane 5, Fig. 5B). The formation of the CArG/SRF complex and the myocardin/CArG/SRF ternary complex was not affected by the addition of Tbx5 proteins (lanes 6 and 8, Fig. 5B), suggesting that TBX5 has no direct effect on the association of myocardin to the SRF/CArG complex.

### Tbx5 mutation abolishes the synergy with myocardin

**Figure 6:**
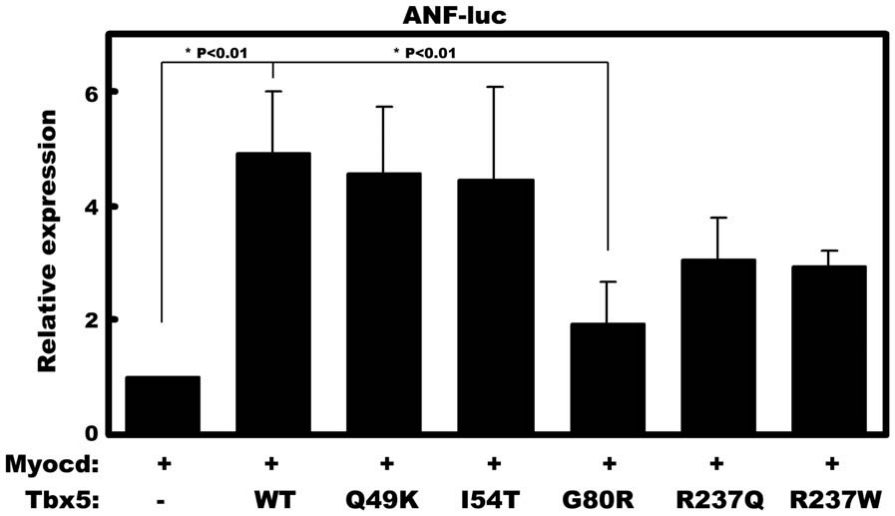
The Tbx5 G80R mutant abolished the synergy between Tbx5 and myocardin to activate cardiac gene expression. COS-7 cells were transfected with an ANF promoter luciferase reporter and expression plasmids for myocardin, Tbx5 or indicated Tbx5 mutants and luciferase activity measured. Luciferase activity was determined 48 hr after transfection and was presented as relative luciferase activity in which the control was assigned a value of 1. Data represent the mean ± s.d. from at least three independent experiments in duplicate. *P<0.01.

Tbx5 has been identified as the Holt-Oram syndrome (HOS) causative gene. Multiple missense mutations of the human Tbx5 gene have been identified from individuals with HOS [4], [40]. Many of these mutations were shown to affect the function of the Tbx5 protein, including the reduction of its transcriptional activation ability and the disruption its interaction with protein co-factors [22]. We tested whether these Tbx5 mutations affect the synergy between myocardin and Tbx5 to activate the ANF promoter. An ANF luciferase reporter construct was co-transfected with expression plasmids encoding myocardin together with wild-type or mutant Tbx5. It has previously been shown that induction of missense mutation into Tbx5 did not affect the expression levels of mutant proteins [22]. Among the mutations tested, Q49K, I54T, R237Q and R237W did not significantly affect their synergy with myocardin. However, the Tbx5 G80R mutation abolished the synergy with myocardin in this assay (Fig. 6).

## Discussion

In this study we demonstrated that Tbx5 and myocardin synergistically activate cardiac gene expression. This activity is specific to cardiac tissue as these two proteins failed to similarly regulate smooth muscle genes. Our results therefore reveal a transcriptional regulatory network for cardiac gene expression.

How do Tbx5 and myocardin cooperate to activate cardiac gene expression? We found that myocardin and Tbx5 interact directly and we mapped their interaction domains to the N-termini of these two proteins. It was known that myocardin does not bind to DNA directly, but instead associates with SRF to form a myocardin/SRF/CArG ternary complex on the promoters/enhancers of its target genes [23], [24], [25]. Tbx5 directly binds to the promoters/enhances of its targets on TBE sites to activate the expression of target genes [4], [9]. Our data indicate that the synergy was primarily dependent on the Tbx5 binding sites TBEs on the ANF promoter. This is consistent with a prior report in which the TBE sites were shown to be required for the synergistic activation of ANF by Tbx5 and Nkx2.5 [9]. Whereas our studies demonstrated that myocardin and Tbx5 physically interact via protein-protein interaction, they did not appear to enhance each other's DNA association on the promoters/enhancers of cardiac genes. These observations suggest that additional transcriptional co-factors are likely involved in the regulation of the binding of myocardin and Tbx5 to target DNAs. It is also possible that the status of chromatin and histone modification influences the binding of these two transcription factors to their targets.

Myocardin is expressed in both cardiac and smooth muscle cells and is capable of activating both cardiac and smooth muscle-specific gene expression. How is the specificity defined? We observed that the interaction between Tbx5 and myocardin helps mediate distinct smooth muscle and cardiac gene expression profiles. In differentiating smooth muscle cells, Tbx5 staining showed that it was present only in the cytoplasm, instead of the nucleus [41]. Therefore the expression of cardiac genes in smooth muscle cells won't be activated by the cooperation of Tbx5 and myocardin; however, the activation of smooth muscle genes by myocardin is not affected. Previous studies suggest that the cellular localization of Tbx5 protein might be controlled by its interaction partners, such as LMP4 [41], [42]. It will be important to determine whether subcellular location of the Tbx5 proteins contributes to its function in the control of cardiac and smooth muscle gene expression.

Tbx5 belongs to the family of T-box containing transcription factors. Several members of this family of transcription factors are also expressed in the heart. Interestingly, Tbx2 was shown to repress the expression of the ANF gene, in part, by impairing the recruitment of Nkx2.5 to the TBE or the NKE [43]. On the other hand, Tbx20 was reported to synergize with Tbx5 to activate cardiac gene expression [44], [45]. Our data demonstrate that Tbx5 and myocardin synergistically activate the expression of cardiac, but not smooth muscle genes. It will be interesting to test whether other members of the Tbx family of transcription factors will also physically and functionally interact with myocardin to regulate cardiac (and smooth muscle) gene expression during development.

Multiple missense mutations of the human Tbx5 gene have been associated with the HOS. Interestingly, these mutations do not uniformly impair the function of Tbx5 in the same manner. For example, Tbx5 mutations G80R and R237Q have different effects on its synergistic transactivation of the cardiac-specific ANF gene, which will likely translate as subtle differences in the phenotype and severity of the HOS [14]. The Tbx5 G80R mutant displayed more severe defect in co-operating with Nkx2.5 to activate the ANF than that of the R237Q mutant [14], [21]. In addition, the synergy of Tbx5 with Sall4 was slightly reduced by mutations Q49K and T54I but dramatically impaired by mutations Q80R and R237W for FGF10 activation [21]. Given the fact that the expression of Tbx5 downstream genes is significantly affected by the dosage of Tbx5 [4], it is reasonable to speculate that the interaction between Tbx5 and myocardin may differentially affect the expression of some genes, like ANF, but not others like SM22. The molecular mechanisms uncovered in this study suggest that the interaction of transcription factors contribute to the expression of their target genes and human disease.

## Materials and Methods

### Plasmids and Constructs

Myocardin and SRF-expression plasmids were described previously [23], [27], [34]. The mouse Tbx5 gene was cloned from a mouse cDNA library and constructed in Myc- and Flag-tagged pCDNA3.1 expression vectors. Myc and Flag-tagged plasmids were used for immuno-precipitation and Western blot experiments. Luciferase reporters under the control of the atrial natriuretic factor (ANF) promoter, truncated ANF promoter, ANF promoter with mutated CArG, 4XCArG, 4XTBE or the SM22 promoter, α-myosin heavy chain (MHC), smooth muscle MHC (SM MHC), as well as the cytomegalovirus lacZ and the Gal4-myocardin construct were as described previously [23], [33], [34], [36]. Gal4-Tbx5 was constructed by inserting the mouse Tbx5 coding cDNA into the EcoRI and XbaI sites of the pM1 vector (Invitrogen). Truncated Tbx5 mutants were generated by PCR with the oligonucleotides for the desired sites in the pMyc-cDNA vector. Tbx5 missense mutants (Q49K, I54T, G80R, R237Q and R237W) were as described previously [22].

### Cell Culture, Transfection and Luciferase Reporter Assays

COS-7 cells were maintained in DMEM with 10% FBS as described [23], [35], [37]. The cells were seeded on plates and transfected the following day with Fugene 6 (Roche) using the protocol provided by the manufacturer. The amount of each type of plasmids was 0.1 μg unless stated otherwise in the text. The total amount of DNA was normalized to the same with the addition of vector DNA. The cells were lysed 48 hrs later and luciferase activity was measured using assay kit (Promega) and the lacZ activity was measured to serve as internal control as described [35]. All experiments were performed in duplicate and repeated at least twice.

### Ethics Statement

Neonatal rat cardiomyocytes were prepared as described [23]. All animal procedures were approved by and performed in accordance with the University of North Carolina Institutional Animal Care and Use Committee under the protocol 08-227.0.

### Co-immunoprecipitation (Co-IP) and Western blot

Co-IP and Western blot analyses were performed as described [23], [35]. Briefly, COS-7 cells in 10-cm plates were transfected with Myc- or Flag- tagged Tbx5 and/or myocardin. After 2 days, the cells were harvested in lysis buffer (20 mM Tris, 150 mM NaCl, 0.5% Triton X-100, 1 mM EDTA, pH 7.5) containing protease inhibitors (Roche) and 1 mM phenylmethylsulfonyl fluoride. The lysates were centrifuged at 14,000x RPM for 10 minutes after sonication for 15 seconds for 3 times. The supernatants were added anti-Myc antibodies and incubated at 4°C for 2 hours, or protein A-agarose beads were added and incubated for 1 more hour. For immunoprecipitation with anti-Flag antibodies, the anti-M2 Agarose beads (Sigma) were added into the cell lysate and incubated for 4 hours at 4°C. Then the beads were spin down and washed with lysis buffer 3 times. The bound proteins were eluted with SDS loading buffer and used for Western blot analysis with anti-Myc (1∶2500; C14, Santa Cruz Biotechnology) and anti-Flag (1∶2500; M2 Sigma) antibodies.

### Electrophoresis mobility shift assay (EMSA)

EMSAs were performed as described previously [35], [36]. The probes were CArG [36] and T-box factor-Binding Element, TBE2 (the up-strand sequence was 5′CTCTTCTCACACCTTTGAAGTGGG3′). The probes were labeled as described [36] and the EMSA procedures were performed as previously described [36].
